# Survival with one versus three centimeters of active decompression during automated head-up CPR in a porcine cardiac arrest model

**DOI:** 10.1016/j.resplu.2026.101231

**Published:** 2026-01-19

**Authors:** Pouria Pourzand, Anja Metzger, Johanna Moore, Bayert Salverda, Hamza Hai, Mithun Suresh, Sarah Bubier, Kerry Bachista, Nicolas Segond, Guillaume Debaty, Keith Lurie

**Affiliations:** a Department of Emergency Medicine, Lehigh Valley Health Network, Bethlehem, PA, USA; b Department of Emergency Medicine, University of Minnesota, Minneapolis, MN, USA; c Hennepin Healthcare Research Institute, Minneapolis, MN, USA; d Department of Medicine, CentraCare-St. Cloud Hospital, St. Cloud, MN, USA; e Mayo Clinic School of Health Sciences, Mayo Clinic in Florida, Jacksonville, FL, USA; f Department of Emergency Medicine, University of Grenoble Alps/CNRS/TIMC-IMAG UMR 5525, Grenoble, France

**Keywords:** Active compression-decompression CPR, Cardiac arrest, HeadUp CPR, Hemodynamics, Impedance threshold device, Survival

## Abstract

**Background:**

Automated head-up (AHUP) CPR, combining controlled head/thorax elevation, active compression-decompression CPR, and an impedance threshold device, has shown improved survival with favorable neurological outcomes versus conventional (C) CPR. The optimal amount of active lift (AD) during AHUP-CPR to optimize survival remains unknown. This study focused primarily on 24-h survival with 1-cm of active lift (AL-1 cm) with a rectilinear waveform versus 3-cm of active lift (AL-3 cm) with a trapezoidal waveform during AHUP-CPR.

**Methods:**

Anesthetized pigs (*n* = 24, ∼40 kg) were randomized to AL-1 cm or AL-3 cm after 10 min of ventricular fibrillation. CPR began with 2 min of C-CPR (21% AP depth, sinusoidal waveform, 100/min), followed by 18 min of AHUP-CPR using the assigned AL. Asynchronous ventilation (10 ml/kg, 10/min) was provided. Epinephrine and amiodarone were administered after 19 min of CPR with defibrillation 1 min later. Primary outcome: 24-h survival; Secondary outcomes: return of spontaneous circulation (ROSC), hemodynamics, epinephrine response, and neurological function (Neurological Deficit Score [NDS], 0 = normal, 320 = death). Statistical analyses included *t*-test, Kaplan-Meier, log-rank, and Mann-Whitney U tests.

**Results:**

ROSC occurred in 6/12 pigs with AL-1 cm vs 12/12 with AL-3 cm (*p* = 0.03), and 24-h survival rates were 16.7% vs 41.7%, respectively (*p* = 0.04). Hemodynamics, ETCO2, epinephrine response, and changes in rSO2 values were significantly higher with AL-3 cm. NDS was 286 ± 79 (AL-1 cm) vs 213 ± 130 (AL-3 cm, *p* = 0.09).

**Conclusion:**

24-h survival rates were significantly higher with AL-3 cm vs AL-1 cm during AHUP-CPR. Together with improved hemodynamics observed with AL-3 cm, these outcomes underscore the critical importance of AL-3 cm to optimize AHUP-CPR.

## Introduction

Out-of-hospital cardiac arrest (OHCA) affects millions of people worldwide each year. On average ≤8% survive with intact neurological function.^1^ The likelihood of surviving with normal cognitive function relies on several factors, including CPR quality, technique, timing, cause of the arrest, and existing underlying conditions.^2^, ^3^, ^4^

Conventional CPR (C-CPR) restores only 15–25% of normal blood flow to vital organs with manual compressions, underscoring the pressing need for better methods to enhance circulation during resuscitation efforts.^5^, ^6^, ^7^ One approach, automated head-up positioning CPR (AHUP-CPR), combines active compression-decompression (ACD) CPR, an impedance threshold device (ITD), and automated gradual head and thorax elevation.^7^, ^8^, ^9^ This approach enhances venous return to the heart by lowering intrathoracic pressure, thereby promoting greater blood flow to the heart and brain, and by lowering intracranial pressure, thereby reducing harmful increases in intracranial pressure generated with chest compressions.^7^, ^9^ Research has demonstrated that AHUP-CPR improves cerebral perfusion pressure (CerPP), cerebral blood flow, end tidal CO2 (ETCO2), regional brain oxygenation (rSO2), and coronary perfusion pressure (CorPP), while also enhancing neurologically intact survival in both animal models and clinical research.^10^, ^11^, ^12^, ^13^, ^14^, ^15^, ^16^, ^17^, ^18^, ^19^, ^20^, ^21^, ^22^

CPR methods have steadily advanced through ongoing research and technological advancements, with a growing focus on enhancing CPR quality and clinical outcomes.^23^ One aspect of AHUP-CPR, the amount of active lift (AL) after each compression, has recently been studied in a porcine model of cardiac arrest. Loss of AL during prolonged AHUP-CPR was associated with a sudden decrease in brain and heart perfusion pressures, whereas incremental restoration of AL was associated with significant step-by-step improvements in hemodynamics, including restoration of ventricular stroke volume.^24^ Another component of CPR, the specific dimensions of the compression waveform, has also been investigated.^25^, ^26^ Research on compression rate, depth, decompression excursion, ACD duration, force, and waveform shape has highlighted that up to 4 cm of AL, as well as faster compression and decompression velocity using a trapezoidal ACD-CPR waveform, may be important to achieving optimal circulation and better clinical outcomes during CPR.^27^, ^28^, ^29^, ^30^

Building on these observations, in this preclinical study, we tested the hypothesis that 24-h survival rates would be significantly higher during AHUP-CPR with AL-3 cm and a trapezoidal waveform with a fast compression and decompression velocity versus AHUP-CPR with AL-1 cm and a rectilinear waveform, with the aim of optimizing CPR mechanics to improve survival outcomes. Planned secondary endpoints included hemodynamics, ETCO2, rSO2, and 24-h neurological survival scores.

## Methods

The study protocol received approval from the Institutional Animal Care Committee at Hennepin Healthcare Research Institute (HHRI) and adhered to the National Research Council's 2011 Guidelines for the Care and Use of Laboratory Animals. Guideline compliance was monitored by a certified and licensed HHRI veterinarian.

The animal preparation and surgical procedures followed previously established methods.^21^, ^31^ Castrated male and female ∼40 kg adult Yorkshire farm pigs were acclimated and fasted overnight prior to the study. On the morning of the study, pigs first received intramuscular ketamine, and then were anesthetized with isoflurane by mask. They were then intubated with a 7.5 mm cuffed endotracheal tube, and mechanical ventilated with a tidal volume of 10 ml/kg (Narkomed, North American Dräger). O2 saturation and ETCO2 levels were monitored (CO2SMO Plus1, Novametrix Systems) and kept within normal physiological levels (>92% and 38–42 mmHg). Femoral arterial and venous access were obtained, and micromanometer-tipped catheters (Mikro-Tip Transducer, Millar Instruments) were used to measure pressures in the descending thoracic aorta (AoP) and right atrium (RAP), respectively. To maintain RA pressure between 7 mmHg and 10 mmHg, maintenance fluids with up to 1000 ml of normal saline were administered during the preparatory phase. The temperature measured with an esophageal probe (TSD202F, BioPac Systems, Inc.) was maintained between 36.5°C and 38.5°C. Regional cerebral tissue perfusion (rSO2) was assessed using near-infrared spectroscopy (Nonin – Sensmart Model X-100, Nonin Medical Inc.). Heparin (5000 units) was administered intravenously during the preparatory phase, followed by 1000 units each hour throughout the study to prevent coagulation. AoP, RAP, surface ECG, rSO2, and ETCO2 data were continuously recorded (BioPac Systems, Inc.).

After completing the surgical preparation and collecting baseline data, fluoroscopy was used to identify and mark the mid-cardiac location for chest compressions in all animals. Ventricular fibrillation (VF) was then induced using a pacing wire (Blazer II, Boston Scientific, Marlborough, MA), which was placed inside the right ventricle under fluoroscopy, three minutes after discontinuation of isoflurane. The study was not blinded. Animals were randomly assigned after induction of VF to their treatment group regardless of sex or weight. After 10 min of untreated VF, CPR was initiated using an automated CPR device specifically designed for swine, as described previously.^24^ The CPR device allows for control of compression and decompression rate, depth, velocity and force to achieve the waveform based on the randomized group. Chest compressions were delivered at 100 per minute with a 50% duty cycle. Over the first 90 s of CPR, compression depth was increased from 19% to 21% of the anteroposterior diameter, reaching a depth of ∼5.0 cm, and it was maintained for the remainder of the protocol.

**Fig. 1:**
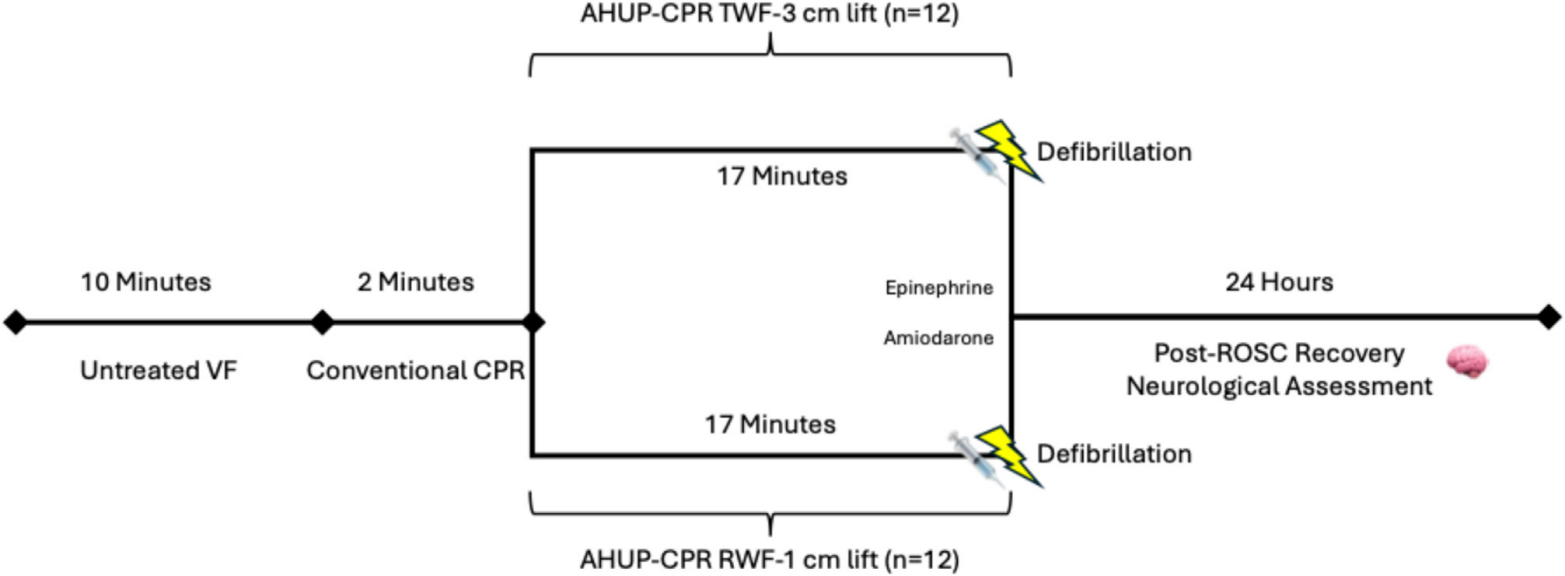
**Schematic of experimental protocol**. *Abbreviations:* VF: ventricular fibrillation; CPR: cardiopulmonary resuscitation; AHUP-CPR: automated head-up CPR; TWF: trapezoidal waveform; RWF: rectilinear waveform; ROSC: return of spontaneous circulation.

As outlined in Fig. 1, the study protocol was initiated with C-CPR using a sinusoidal waveform in a flat and supine position for two minutes, with passive chest wall recoil and no AL. Based on the randomization group, pigs were then treated with ACD + ITD CPR using either AL-1 cm with a rectilinear waveform or AL-3 cm with a trapezoidal waveform, together with an ITD attached to the endotracheal tube with 16 cmH20 of inspiratory resistance (ResQPOD-16, ZOLL Medical, Chelmsford, MA) with the head and thorax elevated to 10 cm and 8 cm, respectively, using a customized controllable porcine head and thorax elevation device. After 2 min at this height, the head and thorax were gradually elevated over another 2-minute period during AHUP-CPR to a maximal height of 24 cm and 9 cm, respectively. After a total of 19 min of CPR, animals received intravenous epinephrine (0.5 mg) and amiodarone (50 mg) and were defibrillated one minute later using a 360 J biphasic shock (Lifepak 15, Physio Control). If sustained return of spontaneous circulation (sROSC) was not achieved, AHUP-CPR was continued, with up to two additional rounds of medication and defibrillation.

Throughout the protocol, positive pressure ventilations were delivered asynchronously using a customized automated resuscitator bag compressor, as previously described.^21^, ^24^, ^32^ A tidal volume of approximately 10 ml/kg of oxygen-enriched room air gas was delivered 10 times/min with 1 s/breath ratio to maintain oxygen saturation >90%. To prevent gasping, a 100 mg IV bolus of succinylcholine (2.5 mg/kg) was administered 30 s after the start of CPR. Arterial blood gases (ABG) were obtained during baseline, before VF, at 10 and 19 min after starting CPR, and hourly in those pigs with a sROSC.

### Post-ROSC care

All animals with sROSC remained anesthetized with isoflurane for four hours, and then they were weaned off the anesthetic and then the ventilator. Once stable, pigs were returned to their holding pens, observed for two hours to ensure they maintained an oxygen saturation above 90%, could rest comfortably in the prone position, and exhibited no signs of distress or seizures, following the guidelines set by the IACUC. All animals that achieved sROSC received subcutaneous buprenorphine and intramuscular flunixin for pain relief. Neurological function was assessed 24 h later by an independent HHRI veterinarian, blinded to treatment groups, using a comprehensive Neurological Deficit Score (NDS).^21^, ^33^ The NDS score ranges from 0, indicating no neurological deficits, to 320, which corresponds to brain death or mortality (NDS score sheet provided in the supplementary file). Following the NDS evaluation, the pigs were sedated and euthanized using intravenous potassium chloride, after which a necropsy was conducted.

### Data analysis

Hemodynamic data were collected during baseline, CPR and after ROSC. AoP, RAP and ETCO2 values were recorded continuously, and 30-s measurements throughout the protocol were averaged and compared between study groups. CorPP was calculated using the mathematical difference between the decompression phase AoP and RAP. The primary outcome was survival up to 24 h post-ROSC. Secondary outcomes included multiple hemodynamic parameters, hemodynamic responsivity to epinephrine at the end of the protocol, maximum rSO2 values and changes in rSO2 over time, sROSC, and 24-h neurological function.

Continuous variables are reported as mean ± SD, with categorical values reported as absolute values or percentages. Statistical analysis was performed using Kaplan-Meier curve analysis, log-rank, and Mann-Whitney t-tests. Mixed-effects linear regression analysis was performed on hemodynamics to evaluate temporal trends and hemodynamic epinephrine responsivity, accounting for repeated measures with random intercepts and slopes for individual animals. Statistical analyses were performed using SPSS version 26 (IBM Corp., NY, USA). A *p*-value of less than 0.05 was considered statistically significant.

## Results

A total of 24 animals were randomized during the period of untreated VF to receive either AL-1 cm (*n* = 12, 8 females and 4 males) or AL-3 cm (*n* = 12, 5 females and 7 males) during AHUP-CPR. Although not statistically significant, male pigs had lower survival rates and poorer neurological function compared to females. In the AL-3 cm group, four of five females survived, with most exhibiting normal or near-normal neurological function. In contrast, only one of seven males in this group survived and demonstrated limited neurological recovery. Similarly, in the AL-1 cm group, two of eight females survived, both showing partial neurological recovery, whereas none of the four males survived.

The mean ± SD of total upward forces applied to achieve 1 cm and 3 cm lift were 27.6 ± 13.7 N and 53.0 ± 12.8 N, respectively. In the AL-1 cm group, the compression and decompression distances were 4.6 cm ± 0.1 cm and 1.3 cm ± 0.2 cm, respectively. In the AL-3 cm group, the compression and decompression distances were 4.6 cm ± 0.2 cm and 3.3 cm ± 0.2 cm, respectively. Airway pressures were measured after each positive pressure ventilation as a surrogate for intrathoracic pressure (ITP). The intrathoracic vacuum was greater in the AL-3 cm versus AL-1 cm group as previously reported.^24^ For example, negative ITP measured after 10 min of AHUP-CPR in 4 consecutive 6 s epochs was −7.5 ± 2.0 mmHg versus −5.6 ± 1.6 mmHg in the AL-3 m versus AL-1 cm groups, respectively (*p* = 0.021).

**Fig. 2:**
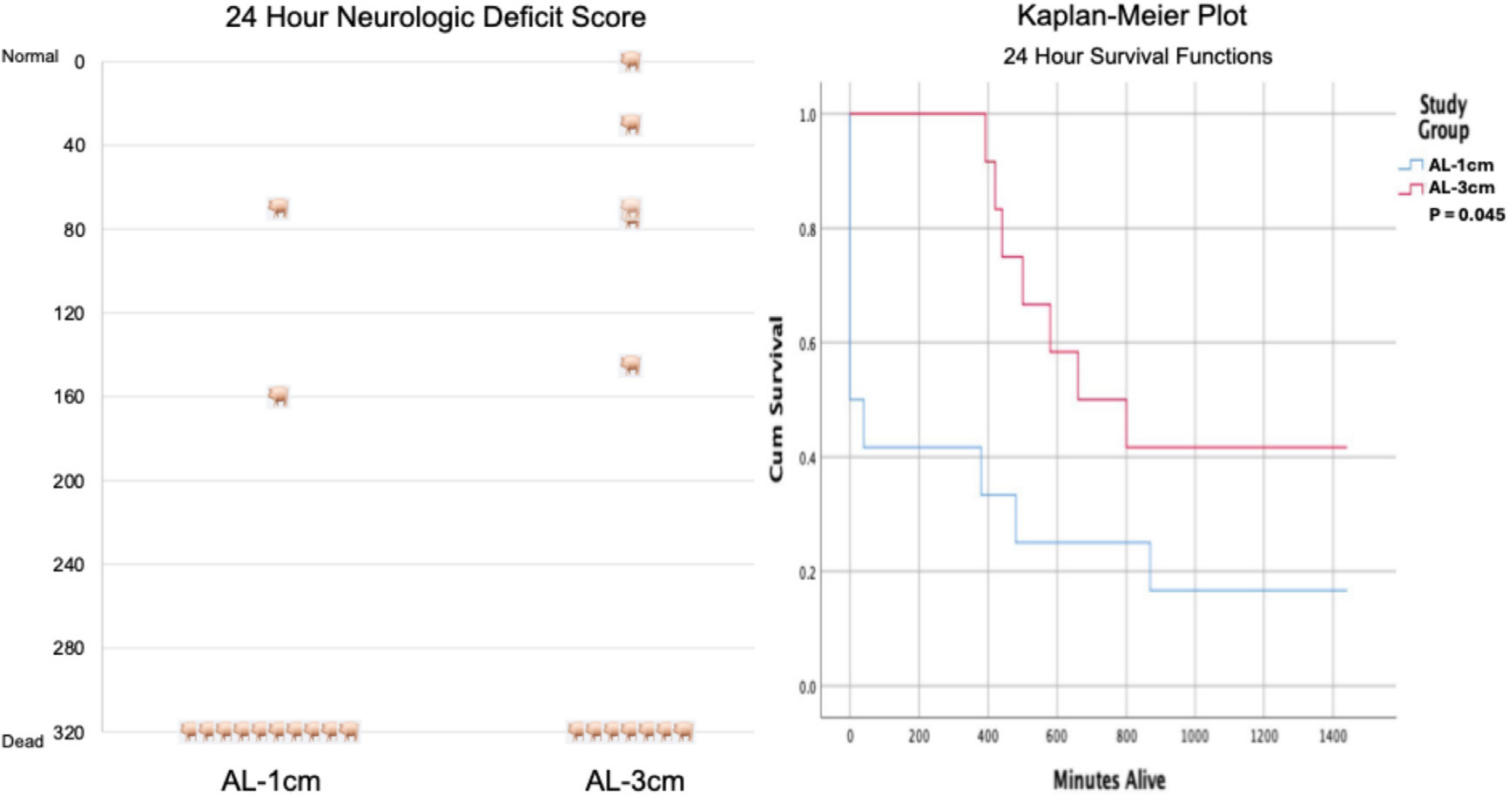
**Comparison of survival with neurological outcomes between automated head-up CPR rectilinear waveform with 1 cm of active lift (AL-1cm) and automated head-up CPR trapezoidal waveform with 3 cm of active lift (AL-3 cm). Mann-Whitney and log-rank tests were used to compare neurological function and cumulative survival rates 24 h after the CPR protocol**.

There were no statistically or clinically significant differences in baseline hemodynamics between the two groups (Supplementary data). sROSC was achieved in 6/12 (50%) pigs treated with AL-1 cm versus 12/12 (100%) treated with AL-3 cm (*p* = 0.03). The primary study outcome, survival up to 24 h, was determined using a Kaplan-Meier curve analysis (Fig. 2). Survival was significantly higher in the AL-3 cm treatment group (*p* = 0.045), with an average 24-h survival rates of 16.7% with AL-1 cm treatment and 42.7% with AL-3 cm treatment, respectively. Neurological outcomes trended higher with AL-3 cm, but the differences did not reach statistical significance, as also shown in Fig. 2. The NDS was 286 ± 79 with AL-1 cm vs 213 ± 130 with AL-3 cm (*p* = 0.09). Of the AL-1 cm 24-h survivors, 1/2 had normal or a modest reduction in consciousness and could stand or walk, whereas 4/5 of AL-3 cm 24-h survivors had normal or a modest reduction in consciousness and could stand and walk. Both groups had similar numbers of complications, including rib and sternal fractures (2.4 ± 1.9 vs 3.1 ± 1.5, respectively). The most common cause of death between sROSC and 24 h was unremitting seizure activity, which could not be suppressed with midazolam. A total of 3 animals post sROSC in the AL-1 cm group and 3 animals in the AL-3 cm group developed drug-refractory central nervous system seizure activity. Per the protocol and IACUC requirements, animals that developed seizures after sROSC, which could not be terminated with midazolam therapy, were euthanized.

**Fig. 3:**
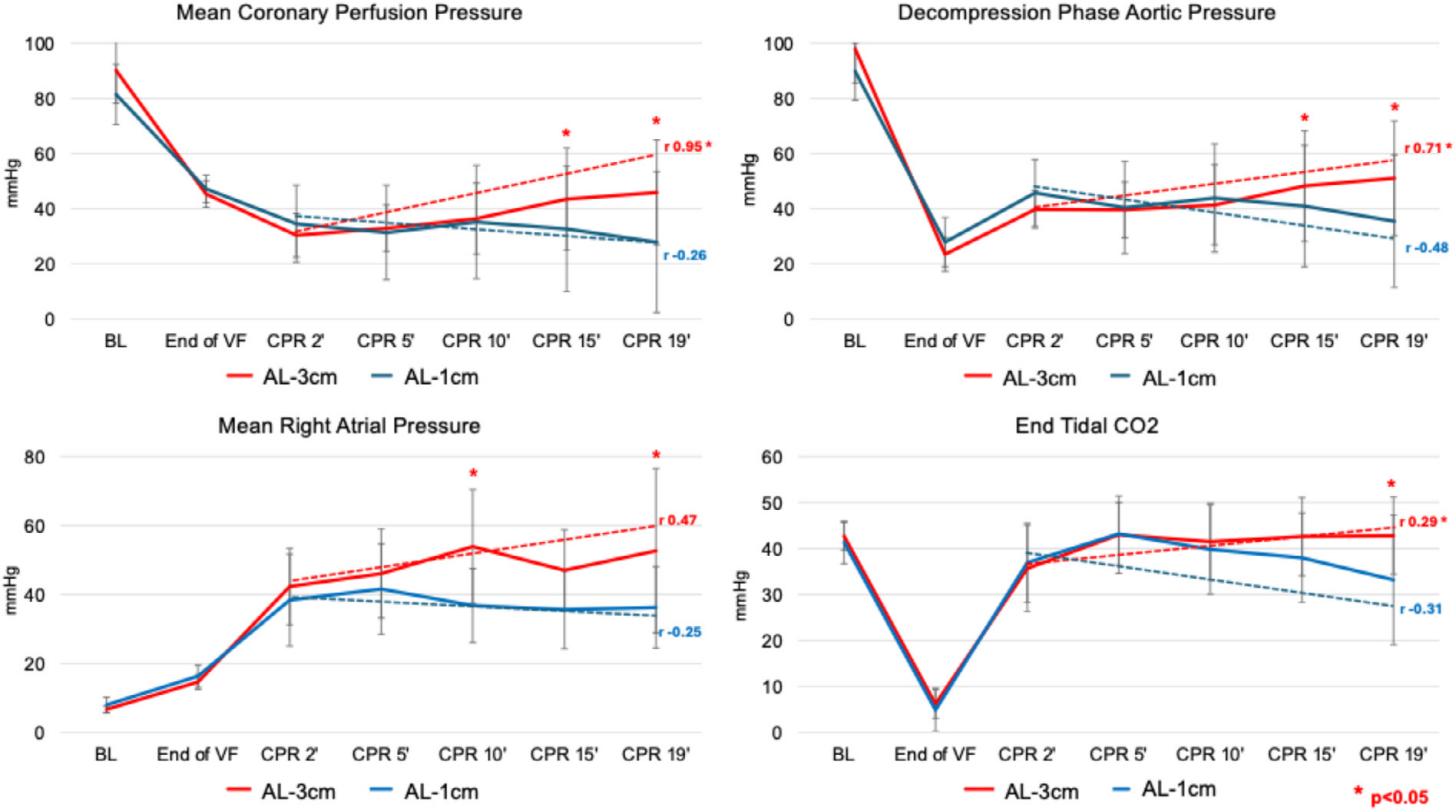
**Comparison of key hemodynamics between automated head-up CPR rectilinear waveform with 1 cm of active lift (AL-1 cm) and automated head-up CPR trapezoidal waveform with 3 cm of active lift (AL-3 cm). A mixed-effect linear regression model was used to compare average values and temporal trends between AL-1 cm and AL-3 cm**. *Abbreviations:* BL: baseline; VF: ventricular fibrillation; CPR: cardiopulmonary resuscitation.

Several key hemodynamic parameters were also observed to be better in the AL-3 cm group. Specifically, mean decompression phase AoP, RAP, ETCO2 and CorPP values were significantly higher with AL-3 cm after about 15 min of CPR (*p* < 0.05). Additionally, as shown in Fig. 3, during AHUP-CPR, the rate of increase in the decompression phase AoP, CorPP and ETCO2 was significantly higher in the AL-3 cm versus AL-1 cm groups.

The difference in RA peak to trough values were also measured. RA compression mean – RA decompression mean values were 111.7 ± 32.1 vs. 79.6 ± 38.6 comparing AL-3 cm versus AL-1 cm, respectively (*p* = 0.046). In addition, the RA maximum compression values – RA minimum decompression values were 191.7 ± 48.8 vs. 117.7 ± 45.6 comparing AL-3 cm versus AL-1 cm, respectively (*p* = 0.001).

**Fig. 4:**
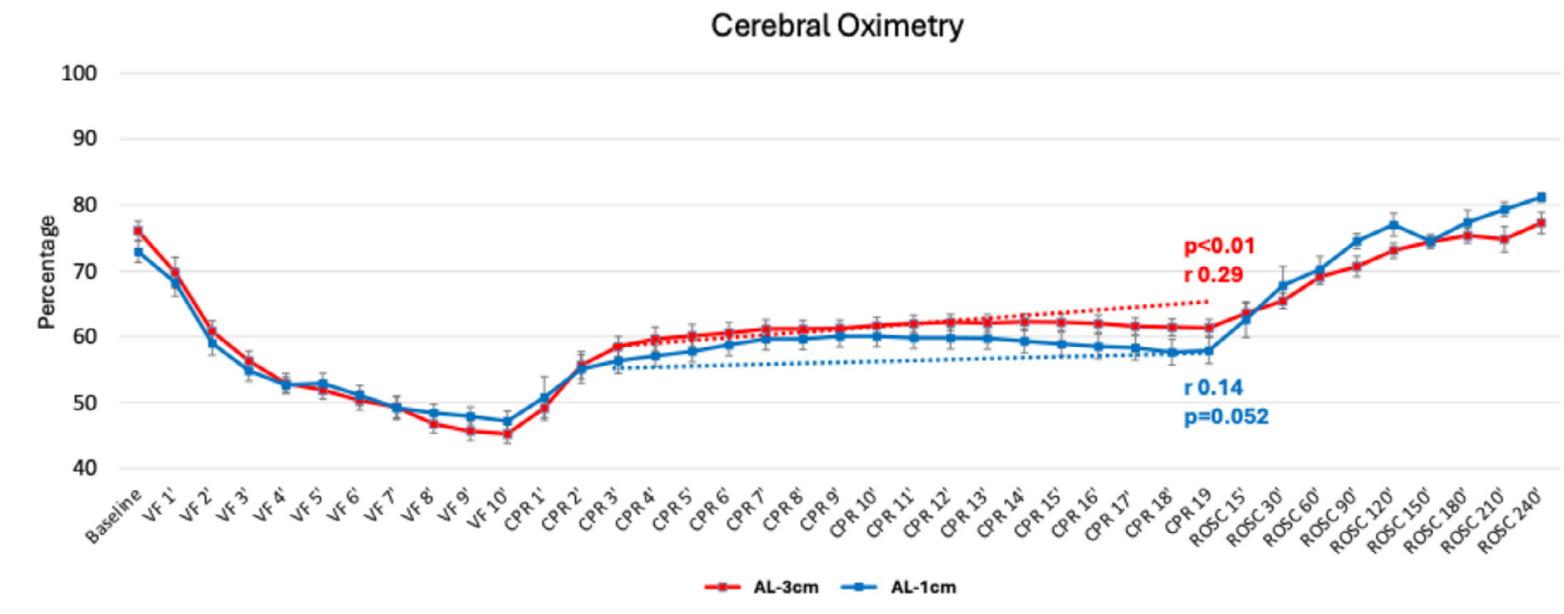
**Cerebral oximetry values at baseline, throughout and after the CPR protocol. A mixed-effect linear regression model with linear confidence was used to compare average values and temporal trends between AL-1 cm and AL-3 cm**. *Abbreviations:* VF: ventricular fibrillation; CPR: cardiopulmonary resuscitation.

**Fig. 5:**
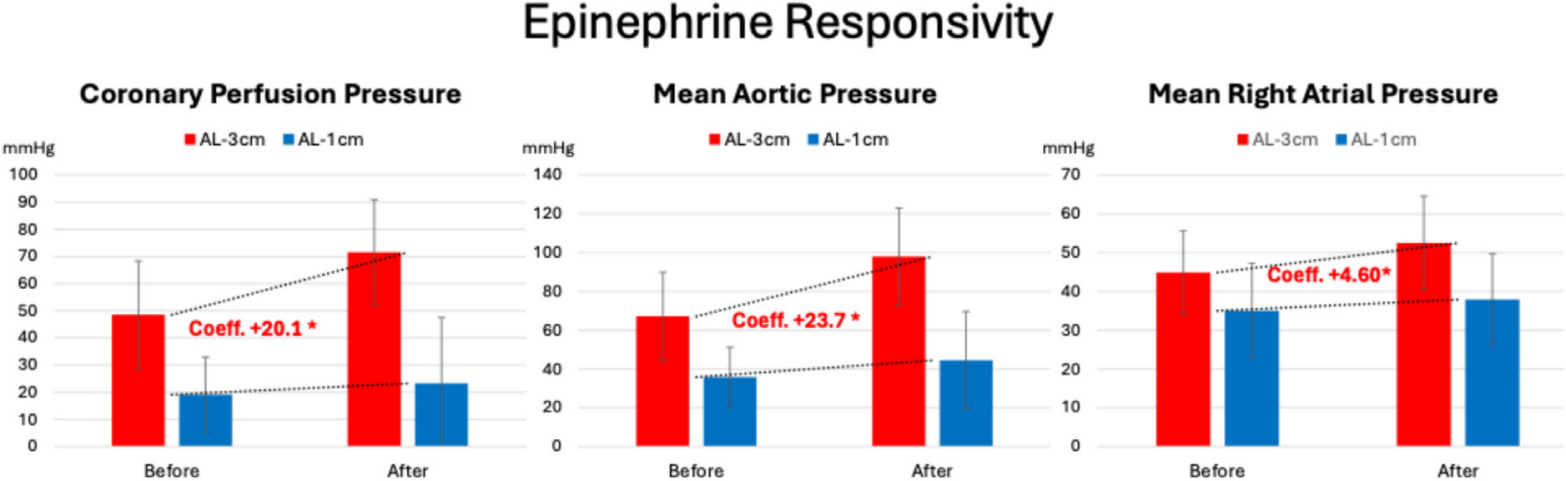
**Key hemodynamics response to epinephrine administration. A mixed-effect linear regression model was used to compare average values and rate of increase (coefficient) between the automated head-up CPR trapezoidal waveform with 3 cm of active lift (AL-3 cm)**.

Other secondary endpoints also supported the benefit of AL-3 cm versus 1 cm-AL. For example, while rSO2 increased in both groups, the rate of increase was significantly higher with AL-3 cm (0.29% per minute, *p* < 0.01) compared with the smaller and non-significant increase with AL-1 cm (0.15% per minute, *p* < 0.052), as shown in Fig. 4. This trend was more pronounced toward the end of the CPR duration (15–19 min). In addition, animals randomized to AL-3 cm showed a significantly heightened responsivity to epinephrine administration after 19 min of continuous CPR, as determined by increased AoP, RAP, and CorPP values assessed one minute later (Fig. 5 and supplementary Table 3). Greater epinephrine responsivity was also associated with a higher likelihood of achieving sROSC, as previously reported.^21^

It is noteworthy that there were no major differences in arterial blood gases between groups (see Supplementary data).

## Discussion

Results from this study demonstrated that AHUP-CPR with active decompression of 3 cm beyond the neutral chest position using a trapezoidal ACD waveform significantly improved 24-h survival, the primary study endpoint, when compared with AHUP-CPR with AL-1 cm using a rectilinear waveform. Key hemodynamic markers of circulation, sROSC rates, and epinephrine responsivity were also significantly higher with AL-3 cm. Differences in neurological outcomes 24-h sROSC did not achieve statistical significance but trended higher with AL-3 cm, with 4/12 pigs versus 1/12 pigs with AL-1 cm surviving with favorable brain function *p* = 0.09.

Building on prior animal studies,^21^, ^24^, ^31^ results from the current study provide strong support for the need for an automated ACD-CPR device capable of actively lifting the chest above the neutral plan by 3 cm during AHUP-CPR. The study protocol was design as a severe prolonged cardiac arrest model, with 10 min of untreated VF and a total of 20 min of cardiac arrest, to mimic real life scenarios of refractory cardiac arrest which may have affected outcomes in both groups. At the time of this writing, no automated suction cup-based clinical automated CPR device is commercially available with >1.5 cm of active lift and the most used automated CPR device only provides 1 cm-AL (LUCAS 3.1). These findings support the possibility that survival rates could be substantially improved further if an automated ACD-CPR with full lift or 3 cm was commercially available for the performance of AHUP-CPR.

Prior studies have demonstrated that incomplete chest recoil impairs circulation,^34^, ^35^ whereas actively lifting the chest to or above the neutral position enhances hemodynamic and survival outcomes.^36^, ^37^, ^38^ Active decompression during CPR with an ITD increases negative intrathoracic pressure, reduces right atrial and ventricular decompression pressures, and increases venous return and cardiac output.^32^, ^36^, ^37^, ^38^, ^39^ When AL-3 cm during ACD-CPR is combined with an ITD, these effects are further amplified, leading to improved perfusion, circulation, and 1-year survival rates.^12^, ^24^, ^40^ More recent studies have shown that active chest wall lift above 1-cm during active decompression optimizes the physiological benefits of AHUP-CPR, by further increasing ventricular filling and cardiac output.^24^, ^32^ As shown previously, using catheters that measured intraventricular pressures and volumes as well as central hemodynamics, each additional centimeter of active lift above the neutral plane contributed to improved outcomes, highlighting the clinical utility of and critical need for greater active lift during AHUP-CPR.^24^

The current study also suggests that the ACD CPR waveform shape may play an important role in optimizing circulation.^3^, ^23^, ^25^, ^41^ Recently, alternative waveforms beyond the traditional sine or square wave forms with constant rate and depth have been studied.^25^, ^26^ Multiple investigations have explored the complex interplay between compression force, depth, duration, and velocity to identify optimal waveforms when performing C-CPR in the flat position.^28^, ^30^, ^42^, ^43^, ^44^, ^45^, ^46^ Among these, sinusoidal, rectilinear, and trapezoidal waveforms have been examined, with evidence suggesting that a trapezoidal waveform with higher compression-decompression velocity enhances hemodynamics.^29^, ^30^, ^47^, ^48^ For that reason, in the current study, we used a trapezoidal waveform in the AL-3 cm group instead of a sinusoidal waveform, which our research laboratory has typically used. This change was made based upon pilot hemodynamic studies comparing AL-3 cm using several different waveform morphologies (data not published). The trapezoidal waveform appeared to result in better hemodynamics, consistent with recent reports by others.^27^, ^28^, ^29^, ^30^, ^47^, ^48^ At present, we do not know how much the change in waveform shape in the current study may have also contributed to the marked survival benefit observed in the AL-3 cm group, in addition to the increased amount of AD. Notably, the piston was positioned at the same optimum position in both experimental groups at baseline and animals were fastened to the treatment table to prevent any significant migration in piston position.^49^

This study had some potential limitations. The study was performed on a relatively small sample size which was calculated to show a potential difference in 24-h survival rates, not neurological function at 24 h. ROSC rates were markedly different between groups (100% versus 50%), and 24-h survival rates, the primary outcome, did achieve statistical significance with a p value of 0.045. The differences in favorable neurological survival were not statistically significance, with a p value of 0.09. However, post-ROSC duration was limited to only 24 h without additional benefit from therapeutic hypothermia or a more extended period for recovery, which could further improve neurological outcomes. Also, epinephrine was administered late in the protocol to reduce its potential effect as a cofounding variable. We speculate that the AL-3 cm group may have had even better outcomes with earlier epinephrine administration. Although not utilized in the current study, we have studied neuroimaging findings in animals treated with AHUP-CPR,^50^ and future studies using neuropathology investigations could add to the current body of evidence demonstrating neuroprotective benefits of AHUP-CPR. Also, healthy young swine with no comorbidities were used in this study, which differs from the actual population of patients that typically experience cardiac arrest. In addition, the etiology of the cardiac arrest was VF. It is not known if results would be similar with other etiologies for cardiac arrest. Lastly, previous studies from our laboratory have shown a marked benefit with progressive amounts of active lift using a sinusoidal waveform.^24^, ^51^ The current study was not designed to prove superiority of a trapezoidal versus rectilinear waveform but rather the superiority of AL-3 cm with a trapezoidal waveform versus AL-1 cm with a rectilinear waveform. Further research is warranted to explore the relative benefits and potential synergies of different amount of active lift with different ACD waveforms.

## Conclusion

This study demonstrated the fundamental importance of actively lifting the anterior chest wall above the neutral position during AHUP-CPR. Hemodynamics, epinephrine responsivity, sROSC rates, and survival to 24-h rates were all significantly higher with AL-3 cm vs AL-1 cm during AHUP-CPR. These outcomes, together with improved ETCO2 and cerebral oximetry values with 3 cm-AL and a trend towards increased neurologically-favorable 24-h survival, underscore the importance of optimizing active decompression during AHUP-CPR.

## CRediT authorship contribution statement

**Pouria Pourzand:** Writing – review & editing, Writing – original draft, Investigation, Formal analysis, Data curation. **Anja Metzger:** Writing – review & editing, Validation, Resources, Project administration, Methodology, Investigation, Funding acquisition, Conceptualization. **Johanna Moore:** Writing – review & editing, Supervision, Project administration, Methodology, Investigation, Funding acquisition, Data curation, Conceptualization. **Bayert Salverda:** Writing – review & editing, Writing – original draft, Formal analysis, Data curation. **Hamza Hai:** Data curation. **Mithun Suresh:** Writing – review & editing, Data curation. **Sarah Bubier:** Data curation. **Kerry Bachista:** Writing – review & editing. **Nicolas Segond:** Writing – review & editing. **Guillaume Debaty:** Writing – review & editing. **Keith Lurie:** Writing – review & editing, Writing – original draft, Visualization, Validation, Supervision, Resources, Project administration, Methodology, Investigation, Funding acquisition, Data curation, Conceptualization.

## Funding

This study was funded by NIH grant #R43HL162179.

## Declaration of competing interest

We confirm that this work is original and has not been submitted, nor is it under consideration for publication elsewhere. The source of funding has been declared, and all authors have read and agreed to the final version of the manuscript. Two co-authors, Drs. Keith Lurie and Anja Metzger work, in part, for AdvancedCPR Solutions, the company that makes a human patient positioning system for Head Up CPR. While the device used for this porcine research is not one that can be used in humans, the relationship with AdvancedCPR Solutions is a potential conflict of interest. Neither Dr. Lurie nor Dr. Metzger received any funding from Zoll or Stryker for their prior work developing the ACD + ITD CPR adjuncts. Also, Nicolas Segond gratefully acknowledges financial support for this publication by the Fullbright Program, which is sponsored by the U.S. Department of State, the Franco-American Commission - Fullbright France and Grenoble-Alpes University. Its contents are solely the responsibility of the author and do not necessarily represent the official views of the Fullbright Program, the Government of the United States, or the Franco-American Commission. No other co-authors have any financial conflicts of interest.
