# Supplementary material for: Survival with one versus three centimeters of active decompression during automated head-up CPR in a porcine cardiac arrest model
Source: Resusc Plus. 2026 Jan 19;28:101231. doi: 10.1016/j.resplu.2026.101231 (PMC12878586; doi:10.1016/j.resplu.2026.101231)

**Table legends:**

Table 1. Hemodynamics during baseline, ventricular fibrillation and cardiopulmonary resuscitation protocol.

Table 2. Arterial blood gas findings during baseline, cardiopulmonary resuscitation and up to 24 hours after obtaining return of circulation.

Table 3. Hemodynamics responsivity to epinephrine administration using a mixed-effect linear regression model was used to compare average values and rate of increase (coefficient) between the automated head-up CPR trapezoidal waveform with 3cm of active lift.

Table 4. NDS score sheet.

**Glossary:**

AL-1cm: automated head-up CPR rectilinear waveform with 1cm of active lift

AL-3cm: automated head-up CPR trapezoidal waveform with 3cm of active lift

AO: aortic pressure

BE: base excess

Comp: compression phase

CorPP: coronary perfusion pressure

CPR: cardiopulmonary resuscitation

Dec: decompression phase

ETCO2: end-tidal CO2

Hb: hemoglobin

HCT: hematocrit

ITP: intrathoracic pressure

pCO2: partial pressure of carbon dioxide

pO2: partial pressure of oxygen

RAP: right atrial pressure

ROSC: return of spontaneous circulation

rSO2: regional cerebral oximetry

SaO2: oxygen saturation

VF: ventricular fibrillation

**Table 1. Hemodynamics:**

|  |  | Dec ITP Mean | Comp. AO Mean | Dec. AO Mean | Mean AO | Comp. RA Mean | Dec RA Mean | Mean RA | ETCO2 | CorPP Mean | rSO2 |
| --- | --- | --- | --- | --- | --- | --- | --- | --- | --- | --- | --- |
| Baseline | AL-3cm | 2.8 ± 0.5 | 122.3 ± 14.0 | 97.8 ± 12.3 | 105.2 ± 13.6 | 5.8 ± 1.0 | 5.7 ± 1.4 | 6.7 ± 1.0 | 42.7 ± 3.0 | 90.1 ± 11.9 | 76.1 ± 5.2 |
|  | AL-1cm | 3.0 ± 0.3 | 106.1 ± 10.9 | 89.7 ± 10.4 | 99.3 ± 11.6 | 6.6 ± 1.2 | 8.3 ± 1.6 | 8.0 ± 2.2 | 41.3 ± 4.6 | 81.4 ± 10.9 | 72.9 ± 5.7 |
|  |  | Dec ITP Mean | Comp. AO Mean | Dec. AO Mean | Mean AO | Comp. RA Mean | Dec RA Mean | Mean RA | ETCO2 | CorPP Mean | rSO2 |
| End of VF | AL-3cm | 0.0 ± 0.0 | 23.5 ± 6.1 | 23.5 ± 6.1 | 23.5 ± 6.1 | 14.6 ± 2.1 | 14.6 ± 2.1 | 14.6 ± 2.1 | 6.2 ± 3.1 | 9.1 ± 6.1 | 45.3 ± 4.8 |
|  | AL-1cm | 0.0 ± 0.0 | 27.9 ± 8.9 | 27.9 ± 8.9 | 27.9 ± 8.9 | 16.3 ± 3.2 | 16.3 ± 3.2 | 16.3 ± 3.2 | 4.9 ± 4.7 | 11.6 ± 9.7 | 47.2 ± 5.0 |
|  |  | Dec ITP Mean | Comp. AO Mean | Dec. AO Mean | Mean AO | Comp. RA Mean | Dec RA Mean | Mean RA | ETCO2 | CorPP Mean | rSO2 |
| CPR 2' | AL-3cm | -0.7± 0.6 | 69.9 ± 12.9 | 39.8 ± 6.8 | 51.9 ± 7.5 | 95.2 ± 35.0 | 9.4 ± 2.6 | 42.3 ± 11.1 | 35.7 ± 9.4 | 30.4 ± 7.9 | 55.7 ± 7.2 |
|  | AL-1cm | --0.3 ± 0.2 | 82.3 ± 21.8 | 45.7 ± 12.1 | 57.9 ± 13.6 | 84.9 ± 37.9 | 11.1 ± 3.0 | 38.4 ± 13.3 | 36.9 ± 8.6 | 34.6 ± 13.9 | 55.1 ± 7.5 |
|  |  | Dec ITP Mean | Comp. AO Mean | Dec. AO Mean | Mean AO | Comp. RA Mean | Dec RA Mean | Mean RA | ETCO2 | CorPP Mean | rSO2 |
| CPR 5' | AL-3cm | -2.0 ± 0.9 | 73.6 ± 10.7 | 39.6 ± 10.1 | 53.0 ± 8.8 | 101.2 ± 32.7 | 6.5 ± 3.6 | 46.1 ± 12.9 | 43.0 ± 8.4 | 33.0 ± 8.4 | 60.2 ± 5.8 |
|  | AL-1cm | -1.8 ± 1.5 | 75.2 ± 18.3 | 40.4 ± 16.7 | 53.0 ± 15.3 | 92.8 ± 35.7 | 9.1 ± 3.0 | 41.6 ± 13.1 | 43.2 ± 6.8 | 31.4 ± 17.1 | 57.8 ± 5.6 |
|  |  | Dec ITP Mean | Comp. AO Mean | Dec. AO Mean | Mean AO | Comp. RA Mean | Dec RA Mean | Mean RA | ETCO2 | CorPP Mean | rSO2 |
| CPR 10' | AL-3cm | -2.1 ± 0.7 | 78.5 ± 16.5 | 41.4 ± 14.5 | 60.3 ± 17.4 | 116.7 ± 32.2 | 5.0 ± 3.3 | 53.9 ± 16.5 | 41.5 ± 8.3 | 36.4 ± 12.9 | 61.7 ± 4.5 |
|  | AL-1cm | -2.5 ± 1.5 | 72.1 ± 26.3 | 43.9 ± 19.6 | 53.4 ± 21.2 | 121.8 ± 43.7 | 8.7 ± 3.5 | 36.8 ± 10.7 | 39.8 ± 6.9 | 35.2 ± 20.5 | 60.1 ± 5.6 |
|  |  | Dec ITP Mean | Comp. AO Mean | Dec. AO Mean | Mean AO | Comp. RA Mean | Dec RA Mean | Mean RA | ETCO2 | CorPP Mean | rSO2 |
| CPR 15' | AL-3cm | -2.1 ± 0.7 | 78.9 ± 22.4 | 48.2 ± 20.0 | 62.0 ± 18.7 | 102.7 ± 40.2 | 4.7 ± 3.5 | 47.0 ± 11.8 | 42.6 ± 8.5 | 43.5 ± 18.5 | 62.2 ± 4.5 |
|  | AL-1cm | -2.3 ± 1.5 | 84.0 ± 32.4 | 41.0 ± 22.0 | 49.8 ± 23.5 | 83.1 ± 33.5 | 8.3 ± 3.5 | 35.6 ± 11.3 | 38.0 ± 9.7 | 32.7 ± 22.7 | 58.9 ± 6.3 |
|  |  | Dec ITP Mean | Comp. AO Mean | Dec. AO Mean | Mean AO | Comp. RA Mean | Dec RA Mean | Mean RA | ETCO2 | CorPP Mean | rSO2 |
| CPR 19' | AL-3cm | -1.9 ± 0.6 | 83.8 ± 25.8 | 51.0 ± 20.8 | 65.3 ± 21.1 | 111.3 ± 32.1 | 5.1 ± 3.6 | 52.7 ± 23.8 | 42.8 ± 8.4 | 45.9 ± 19.0 | 61.3 ± 4.4 |
|  | AL-1cm | -1.8 ± 1.2 | 44.7 ± 30.5 | 35.5 ± 24.0 | 44.4 ± 26.1 | 82.0 ± 33.1 | 7.6 ± 3.5 | 36.3 ± 11.8 | 33.2 ± 14.1 | 27.9 ± 25.5 | 57.9 ± 6.9 |

**Table 2. ABG**

|  |  | pH | pCO2 | pO2 | HCT | HCO3 | Be Ecf | SaO2 | Hgb |
| --- | --- | --- | --- | --- | --- | --- | --- | --- | --- |
| Baseline | AL-3cm | 7.44 ± 0.03 | 43.5 ± 2.3 | 117.7 ± 14.2 | 22.2 ± 2.2 | 29.4 ± 1.3 | 5.2 ± 1.6 | 98.6 ± 0.6 | 6.6 ± 0.7 |
|  | AL-1cm | 7.43 ± 0.04 | 44.5 ± 3.5 | 104.1 ± 12.9 | 23.8 ± 2.6 | 30.7 ± 3.8 | 5.5 ± 2.9 | 97.9 ± 0.8 | 7.4 ± 0.8 |
| CPR 10' | AL-3cm | 7.14 ± 0.07 | 52.5 ± 9.3 | 73.7 ± 17.5 | 29.9 ± 4.2 | 17.6 ± 1.3 | -11.4 ± 1.6 | 84.8 ± 10.7 | 9.3 ± 1.3 |
|  | AL-1cm | 7.12 ± 0.04 | 52.5 ± 6.7 | 82.5 ± 32.5 | 30.4 ± 5.3 | 17.2 ± 2.1 | -12.1 ± 2.4 | 84.3 ± 12.4 | 9.4 ± 1.7 |
| CPR 19' | AL-3cm | 7.11 ± 0.07 | 53.0 ± 7.6 | 72.8 ± 14.2 | 31.3 ± 4.6 | 16.7 ± 1.8 | -12.9 ± 2.6 | 84.3 ± 10.1 | 9.7 ± 1.4 |
|  | AL-1cm | 7.11 ± 0.06 | 51.5 ± 9.3 | 71.6 ± 24.0 | 32.3 ± 5.0 | 16.3 ± 1.8 | -13.1 ± 2.0 | 80.7 ± 14.0 | 10.0 ± 1.5 |
| ROSC 15' | AL-3cm | 7.07 ± 0.09 | 55.7 ± 8.1 | 122.6 ± 36.9 | 32.8 ± 5.1 | 16.4 ± 2.8 | -13.7 ± 4.0 | 94.3 ± 5.6 | 10.0 ± 1.5 |
|  | AL-1cm | 7.06 ± 0.05 | 58.8 ± 6.7 | 133.8 ± 44.5 | 33.2 ± 3.9 | 16.7 ± 1.3 | -13.6 ± 1.8 | 96.0 ± 2.8 | 10.3 ± 1.2 |
| ROSC 60' | AL-3cm | 7.22 ± 0.11 | 44.9 ± 6.9 | 155.0 ± 59.2 | 30.9 ± 4.4 | 18.4 ± 2.8 | -9.3 ± 4.3 | 97.8 ± 3.1 | 9.6 ± 1.4 |
|  | AL-1cm | 7.25 ± 0.06 | 46.6 ± 5.1 | 203.6 ± 56.0 | 30.6 ± 4.0 | 20.2 ± 0.7 | -7.0 ± 1.6 | 99.4 ± 0.5 | 9.5 ± 1.3 |
| ROSC120' | AL-3cm | 7.27 ± 0.11 | 45.9 ± 7.9 | 128.5 ± 57.1 | 31.2 ± 4.0 | 21.1 ± 3.5 | -5.8 ± 5.0 | 97.8 ± 1.4 | 9.7 ± 1.2 |
|  | AL-1cm | 7.30 ± 0.07 | 44.2 ± 3.7 | 172.4 ± 87.5 | 29.6 ± 3.4 | 21.7 ± 2.3 | -4.8 ± 3.3 | 98.8 ± 0.7 | 9.2 ± 1.1 |
| ROSC 180' | AL-3cm | 7.32 ± 0.10 | 44.2 ± 6.7 | 115.6 ± 19.4 | 30.1 ± 3.7 | 22.9 ± 3.5 | -3.1 ± 4.8 | 97.6 ± 1.9 | 9.3 ± 1.2 |
|  | AL-1cm | 7.34 ± 0.08 | 42.0 ± 3.2 | 106.4 ± 13.6 | 29.6 ± 3.8 | 22.9 ± 2.9 | -2.9 ± 4.1 | 97.4 ± 1.7 | 9.2 ± 1.2 |
| ROSC 240' | AL-3cm | 7.37 ± 0.08 | 42.9 ± 5.8 | 100.6 ± 17.7 | 28.8 ± 3.9 | 24.6 ± 3.3 | -0.7 ± 4.3 | 96.9 ± 2.4 | 8.9 ± 1.2 |
|  | AL-1cm | 7.34 ± 0.09 | 44.0 ± 4.1 | 99.5 ± 16.5 | 30.5 ± 2.3 | 23.9 ± 2.7 | -1.8 ± 4.1 | 96.8 ± 2.3 | 9.5 ± 0.7 |
| ROSC 24 Hr | AL-3cm | 7.44 ± 0.03 | 43.0 ± 3.0 | 132.0 ± 7.0 | 24.0 ± 1.0 | 28.8 ± 0.3 | 4.6 ± 0.8 | 99.0 ± 0.1 | 7.5 ± 0.4 |
|  | AL-1cm | 7.36 ± 0.04 | 43.5 ± 8.5 | 233.5 ± 151.5 | 27.0 ± 2.0 | 24.3 ± 2.6 | 1.2 ± 1.9 | 98.0 ± 2.0 | 8.4 ± 0.6 |

**Table 3. Epinephrine responsivity**

|  |  | Comp. AO Mean | Dec. AO Mean | Mean AO | Comp. RA Mean | Dec RA Mean | Mean RA | ETCO2 | CorPP Mean |
| --- | --- | --- | --- | --- | --- | --- | --- | --- | --- |
| AL-3cm  N = 10 | Before Epinephrine | 87.2 ± 26.6 | 53.5 ± 21.9 | 67.1 ± 22.7 | 107.7 ± 34.0 | 5.0 ± 3.5 | 44.9 ± 10.8 | 42.9 ± 8.8 | 48.5 ± 19.8 |
|  | Before Shock | 113.3 ± 27.3 | 82.5 ± 22.8 | 97.9 ± 25.1 | 93.4 ± 21.0 | 11.1 ± 4.2 | 52.5 ± 12.1 | 39.3 ± 7.7 | 71.5 ± 19.4 |
|  | p value | **0.001** | **<0.001** | **<0.001** | 0.0189 | **<0.001** | **<0.001** | **0.007** | **<0.001** |
|  |  |  |  |  |  |  |  |  |  |
|  |  | Comp. AO Mean | Dec. AO Mean | Mean AO | Comp. RA Mean | Dec RA Mean | Mean RA | ETCO2 | CorPP Mean |
| AL-1cm  N = 10 | Before Epinephrine | 49.8 ± 20.4 | 27.4 ± 14.4 | 35.8 ± 15.4 | 77.9 ± 34.5 | 8.3 ± 3.2 | 35.0 ± 12.3 | 29.9 ± 12.9 | 19.1 ± 13.8 |
|  | Before Shock | 51.8 ± 26.8 | 36.7 ± 23.6 | 44.4 ± 25.2 | 62.6 ± 22.1 | 13.5 ± 4.1 | 37.9 ± 11.8 | 27.2 ± 12.1 | 23.2 ± 24.3 |
|  | p value | 0.553 | 0.325 | 0.058 | **0.010** | **0.007** | **0.006** | **0.008** | 0.669 |
|  | **Coefficience**  **Between**  **AL-1 vs AL-3cm** | **-31.8 *** | **-21.0 *** | **-23.7 *** | **-8.20** | **0.56** | **-4.60*** | **-0.72** | **-20.1 *** |

Table 4. NDS score sheet


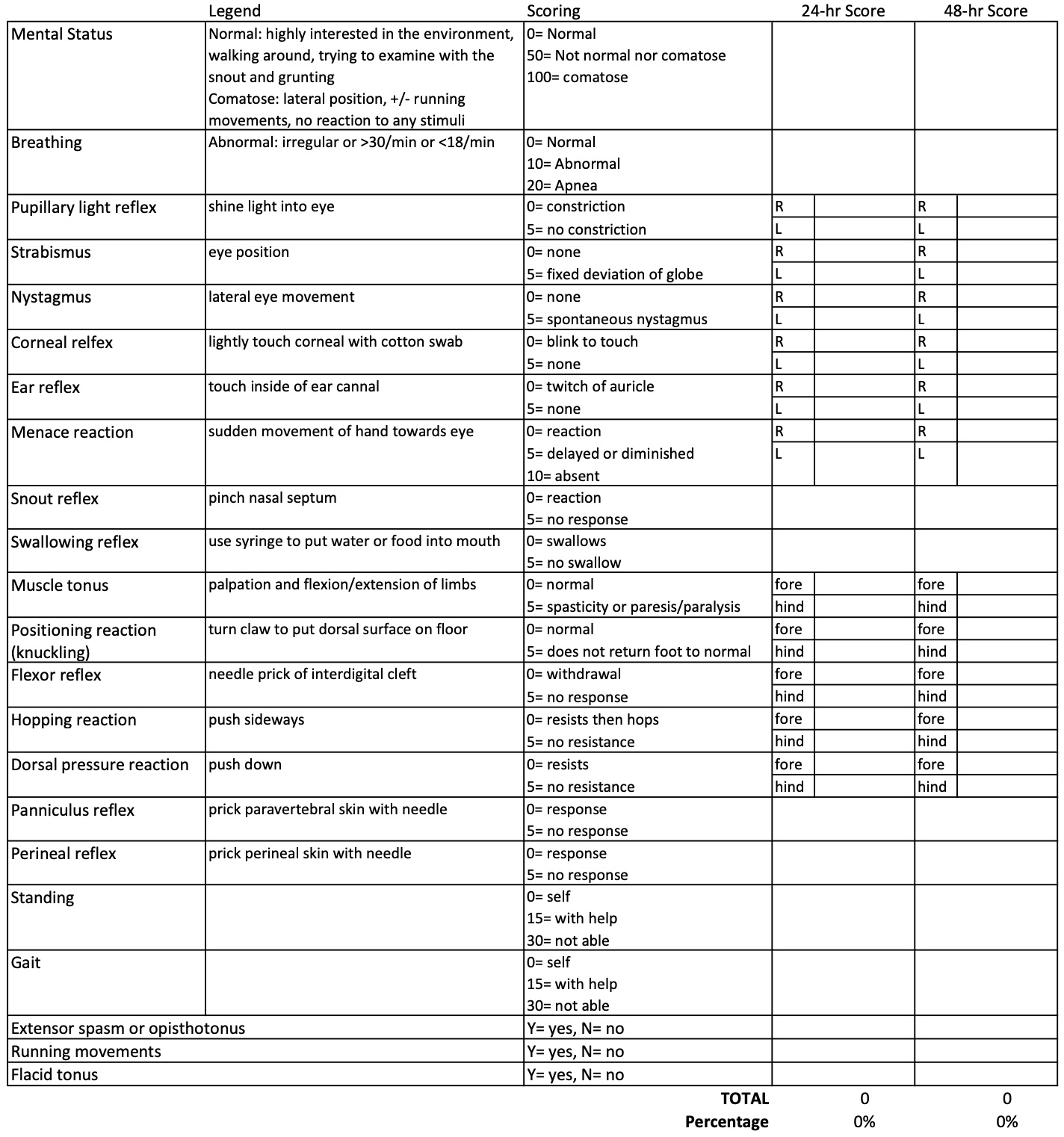

Supplement: Supplementary Data 1 [file mmc1.docx]
